# Whole-Organ analysis of calcium behaviour in the developing pistil of olive (*Olea europaea *L.) as a tool for the determination of key events in sexual plant reproduction

**DOI:** 10.1186/1471-2229-11-150

**Published:** 2011-11-03

**Authors:** Krzysztof Zienkiewicz, Juan D Rejón, Cynthia Suárez, Antonio J Castro, Juan de Dios Alché, María Isabel Rodríguez García

**Affiliations:** 1 Departamento de Bioquímica, Biología Celular y Molecular de Plantas, Estación Experimental del Zaidín (CSIC), Profesor Albareda 1, 18008, Granada, Spain; 2 Department of Cell Biology, Institute of General and Molecular Biology, Nicolaus Copernicus University, Gargarina 9, 87-100, Toruń, Poland

## Abstract

**Background:**

The pistil is a place where multiple interactions between cells of different types, origin, and function occur. Ca^2+ ^is one of the key signal molecules in plants and animals. Despite the numerous studies on Ca^2+ ^signalling during pollen-pistil interactions, which constitute one of the main topics of plant physiology, studies on Ca^2+ ^dynamics in the pistil during flower formation are scarce. The purpose of this study was to analyze the contents and *in situ *localization of Ca^2+ ^at the whole-organ level in the pistil of olive during the whole course of flower development.

**Results:**

The obtained results showed significant changes in Ca^2+ ^levels and distribution during olive pistil development. In the flower buds, the lowest levels of detectable Ca^2+ ^were observed. As flower development proceeded, the Ca^2+ ^amount in the pistil successively increased and reached the highest levels just after anther dehiscence. When the anthers and petals fell down a dramatic but not complete drop in calcium contents occurred in all pistil parts. *In situ *Ca^2+ ^localization showed a gradual accumulation on the stigma, and further expansion toward the style and the ovary after anther dehiscence. At the post-anthesis phase, the Ca^2+ ^signal on the stigmatic surface decreased, but in the ovary a specific accumulation of calcium was observed only in one of the four ovules. Ultrastructural localization confirmed the presence of Ca^2+ ^in the intracellular matrix and in the exudate secreted by stigmatic papillae.

**Conclusions:**

This is the first report to analyze calcium in the olive pistil during its development. According to our results *in situ *calcium localization by Fluo-3 AM injection is an effective tool to follow the pistil maturity degree and the spatial organization of calcium-dependent events of sexual reproduction occurring in developing pistil of angiosperms. The progressive increase of the Ca^2+ ^pool during olive pistil development shown by us reflects the degree of pistil maturity. Ca^2+ ^distribution at flower anthesis reflects the spatio-functional relationship of calcium with pollen-stigma interaction, progamic phase, fertilization and stigma senescence.

## Background

Flower development leads to the formation of functional male and female reproductive organs (i.e., anthers and pistils, respectively). At anthesis, the flower is completely open, anther dehiscence occurs, and pollen grains are released. The progamic phase begins when pollen grains land on the receptive stigma and germinate, forming a pollen tube that grows through the sporophytic tissues of the pistil. Finally, the pollen tube reaches the female gametophyte and releases 2 sperm cells that fuse with the target cells of the embryo sac, allowing double fertilization. The result of this process is the formation of a diploid embryo and a triploid endosperm that constitute the seed. Thus, the pistil is a place where multiple interactions between cells of different types, origin, and function occur [1].

Calcium is present in living organisms as a mixture of free, loosely bound, and bound cations. The different states of Ca^2+ ^are strongly correlated with its activity in cellular metabolism [2,3]. The pool of bound Ca^2+ ^is insoluble and serves mainly as a structural component. The loosely bound Ca^2+ ^pool has lower affinity and is the main form of calcium in most cell types [3]. This pool of Ca^2+ ^is often located in the cell walls and cellular organelles or is associated with specific proteins that use Ca^2+ ^as a coenzyme or regulate Ca^2+ ^concentration [4]. Free Ca^2+ ^is one of the key signal molecules in plants and animals [5] and is involved in multiple signal transduction pathways, which are fundamental for many intercellular and intracellular interactions [6,7].

Calcium plays an essential role in pollen-pistil interactions during the progamic phase [8]. Studies on Ca^2+ ^signalling during pollen tube growth are numerous and constitute one of the main topics of plant physiology [9]. To date, it has been proven that Ca^2+ ^acts as a key factor for proper pollen germination and pollen tube growth, pollen tube guidance, and gamete fusion [10-13]. Thus, it has been demonstrated that growing pollen tubes take up Ca^2+ ^ions from the medium [14], and the Ca^2+ ^ions accumulate in the apical zone of the pollen tube, forming a characteristic tip-to-base gradient [15]. In the pistil, the optimal Ca^2+ ^concentration required for pollen germination is provided by the stigma [16-19]. Most studies concerning the role of Ca^2+ ^in the pistil have been performed at the onset of anthesis [19-22]. Nevertheless, studies on Ca^2+ ^dynamics in the pistil during flower formation are scarce.

Fluorescence imaging of Ca^2+ ^has been extensively applied, mainly in animal cells, by using different fluorescence probes [23]. The most commonly used techniques of loading Ca^2+^-sensitive dyes into plant samples are acid loading, electroporation, and microinjection [24-26]. However, the main limitations of the above-mentioned methods are as follows: (1) a relatively small area of dye application in the sample, which is restricted to single cells, and (2) the presence of esterases, which might potentially hydrolyze the dye esters, in the cell walls [27,28]. So far, the only study on the successful loading of a Ca^2+^-sensitive dye into a whole plant organ was performed by Zhang *et al*. [28]. They analyzed the intracellular localization of Ca^2+ ^in intact wheat roots loaded with the acetoxymethyl ester of Fluo-3.

Up to date there are no reports concerning the calcium behaviour in the olive pistils. The purpose of this study was to analyze the contents and localization of free and loosely bound pools of Ca^2+ ^in the pistil of the olive, from pre- to post-anthesis period of flower development. Previously, we provided a detailed cytological and histological description of the olive pistil tissues [29,30]. The pistil of the olive is composed of a wet stigma, a solid style, and a bilocular ovary with 2 ovules per loculus. However, only one ovule (or two in exceptional cases) is going to be fertilized, since majority of the olive seeds contain only one embryo [31]. We have also reported here the successful injection of the Ca^2+^-sensitive dye Fluo-3 into inflorescences as a useful tool for *in situ *Ca^2+ ^localization in the intact pistils.

## Results

### Experimental design

**Figure 1:**
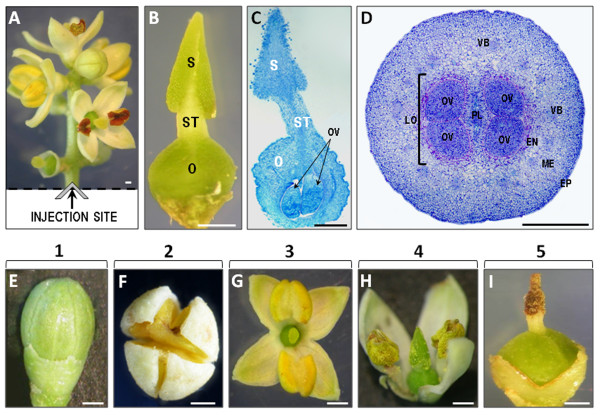
**Experimental design and plant material**. (A) Experimental design: fluorescent Ca^2+ ^indicator was injected directly into the inflorescence peduncle just after it was harvested from the tree. (B) Morphology of the olive pistil harvested from an opening flower (stage 2). (C) Longitudinal section of a mature pistil of an open flower after fixation and methylene blue staining. (D) Transverse section of an ovary from a mature pistil of a flower with dehiscent anthers after fixation and methylene blue staining. **(E-I**) Olive flower developmental stages viewed using a stereomicroscope. (E) stage 1, green flower bud; (F) stage 2, opening flower; (G) stage 3, open flower with turgid yellow anthers; (H) stage 4, open flower with dehiscent anthers; (I) stage 5, flower without anthers and petals, brown stigma, and thick ovary. EN - endocarp, EP - epidermis, ME - mesocarp, O - ovary, OV - ovule, LO - loculus, P - placenta, S - stigma, ST - style, VB - vascular bundles. Bars = 0.5 mm.

*In situ *detection of Ca^2+ ^in olive pistils was carried out by direct injection of the Fluo-3 AM dye into the peduncle of the inflorescence at the site of the cut, as shown in Figure 1A. At each developmental stage, the pistil is composed of a bilobed, wet stigma; a short style; and a round ovary (Figure 1B). The ovary encloses 2 loculi separated by a substantial placenta, and each loculus contains 2 ovules (Figure 1C and 1D). Within the phenologically mixed populations of the flowers, we selected 5 major developmental stages of the olive flower for further experiments (Figure 1E-I): green buds (stage 1; Figure 1E); opening flowers (stage 2; Figure 1F); open flowers with petals recently separated; visible pistil and yellow, turgid, and intact anthers (stage 3; Figure 1G); open flowers with dehiscent anthers (stage 4; Figure 1H); and flowers without anthers and petals (stage 5; Figure 1I).

### Ca^2+ ^content in floral organs during olive flower development

**Figure 2:**
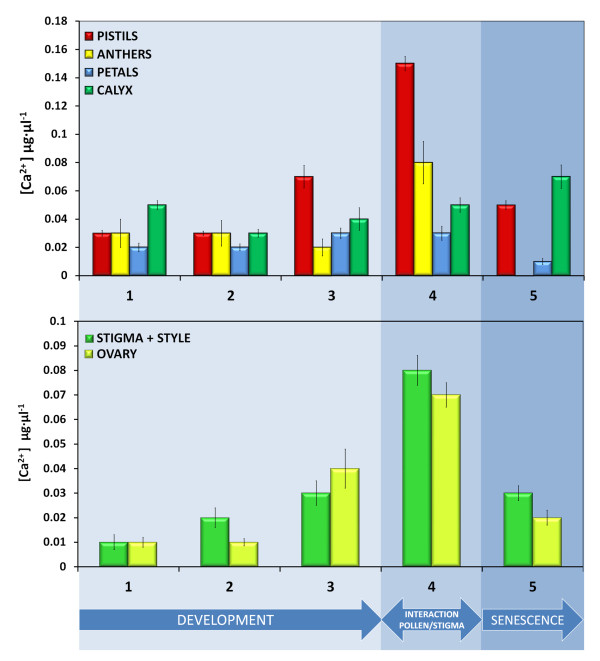
**The Ca^2+ ^content (μg·μl^-1^) of olive floral organs during flower development**. (A) Ca^2+ ^content in the extracts from pistils (black bars), anthers (white bars), petals (light gray bars) and calyx (dark gray bars). Values are mean ± SD values of 3 independent experiments. (B) Comparison of Ca^2+ ^pools from pistil upper parts (stigma with style; black bars) and ovary (light gray bars) at different stages of olive flower development.

To compare the pistil Ca^2+ ^pool in relation to other parts of the flower, we analyzed Ca^2+ ^content during the whole course of olive flower development. The Ca^2+ ^content (μg·μl^-1^) in the extracts of separated floral organs is shown in Figure 2. At the green flower-bud stage (stage 1), pistils, anthers, and petals contained similarly low amounts of Ca^2+^, with exception calyx where calcium levels were slightly higher (Figure 2A). When the sepals turned white (stage 2), the pool of Ca^2+ ^in the analyzed floral organs was similar to that observed in the previous developmental stage (Figure 2A). However, some decrease in the Ca^2+ ^content of the calyx was observed. When the flower was completely open (stage 3), the pistil contained a significantly higher content of Ca^2+ ^than the other floral organs (Figure 2A). In comparison with the previous developmental stages, more than 2-fold increase of the pistil Ca^2+ ^pool was observed at this stage. At the time of anther dehiscence (stage 4), Ca^2+ ^content in the pistil was the highest among all floral organs (Figure 2A). This increase was more than 6-fold in comparison with the green flower bud (stage 2) and more than 3-fold when compared with flower with turgid anthers (stage 3). At this stage of flower development, a significant amount of Ca^2+ ^was also found in the anthers (Figure 2A), whereas in the petals and calyx, there were no significant differences in comparison to stage 3 (Figure 2A). After anther loss (stage 5), a strong decrease in Ca^2+ ^content was shown in the remaining floral organs, except the calyx, which suffered a slight increase in Ca^2+ ^concentration (Figure 2A). For pistil, this decrease was more than 3-fold in comparison with that found in stage 4.

A more detailed analysis of the changes in the olive pistil Ca^2+ ^pool was performed using the separated parts of the pistil: stigma with style and ovary (Figure 2B). At stage 1, the lowest pool of Ca^2+^, with similar amounts of Ca^2+ ^in both pistil parts (stigma with style and ovary), was observed. During flower anthesis (from stage 2 to stage 4), the Ca^2+ ^pool increased progressively and reached the maximal values just after anther dehiscence (stage 4). At the latest analyzed stage (stage 5) a significant decrease of Ca^2+ ^levels was observed in the upper parts of the pistil (stigma and style) and in the ovary (Figure 2B).

### Fluorescence in situ detection of Ca^2+ ^in the olive pistil

**Figure 3:**
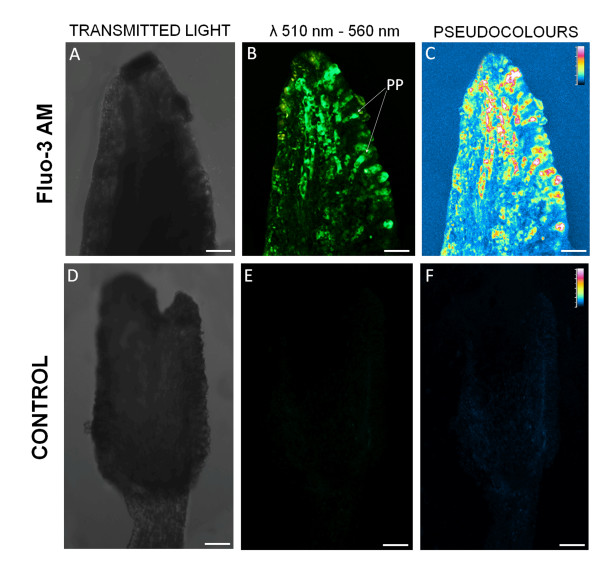
**Confocal images of the pistil injected with Fluo-3 (A-C) and control pistil (D-F)**. Pseudocolor images enhance the visualization of the incorporated Fluo-3 and show the intensity of fluorescence. Minimal fluorescence levels are visible as dark, whereas fluorescence levels of the highest intensity are indicated as white. (A-C) Optical sections of the stigma at stage 3 of flower development after Fluo-3 injection. The signal corresponding to the incorporated Fluo-3 is visualized as green. The highest levels of fluorescence are present in papillae cells (PP). (D-F) In the stigma of the pistil injected with control solution, no green fluorescence is present. Bars = 100 μm.

In order to follow the dynamic of free calcium ions in the olive pistils, the fluorescent indicator Fluo-3 AM was injected directly into the inflorescences. To confirm the presence of the incorporated Fluo-3 AM, we compared the fluorescence emitted by olive pistils from injected peduncles with that of the pistils taken from control peduncles (Figure 3). Detailed analysis under a confocal microscope revealed significant differences between the levels of the signal in pistils treated with Fluo-3 AM and the control. After injection of Fluo-3 AM, green fluorescence was observed on the stigma surface, mostly attached to the papillae cells (Figure 3B-C). Control pistils were practically devoid of green fluorescence (Figure 3D-F).

**Figure 4:**
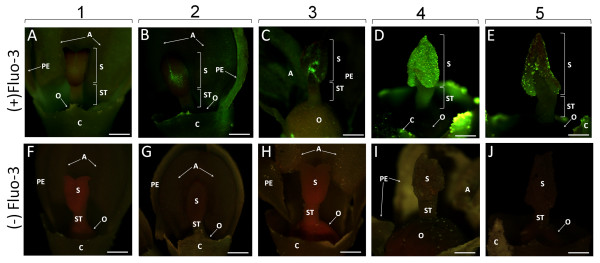
**Detection of Ca^2+ ^by Fluo-3 AM in the pistils during olive flower development**. Images were obtained using a stereomicroscope under blue light (488 nm). Microphotographs in the upper row show the buds/flowers taken from injected inflorescences [(+) Fluo-3], whereas the lower row shows control buds/flowers [(-) Fluo-3] from each corresponding developmental stage. (A) Green flower bud (stage 1): practically no labelling is present in the stigma. (B) White flower bud (stage 2): the labelling appears in some areas of the stigmatic surface. (C) Flower with turgid anthers (stage 3): well-distinguishable green fluorescence is located in the outer part of the stigma. (D) Flower with dehiscent anthers (stage 4): strong labelling is distributed throughout the stigmatic surface. Green fluorescence is also emitted from the stylar tissues. (E) Flower without sepals and petals (stage 5): the labelling is limited to small areas of the stigmatic surface. (F-J) Controls of the examined developmental stages (1-5). No green fluorescence can be detected in any analyzed stage. A - anthers, C - calyx, O - ovary, PE - petals, S - stigma, ST - style. Bars = 0.5 mm.

Initially, Ca^2+ ^distribution in the external parts of developing pistils was analyzed using an epifluorescence stereomicroscope. All the samples analyzed at different stages of olive flower development showed the same fluorescence pattern (Figure 4). The pistil of the green flower bud (stage 1) showed practically no fluorescent signal (Figure 4A). During stage 2, we observed a green signal located only in some areas of the stigmatic surface (Figure 4B). In the open flower with turgid anthers (stage 3), the green fluorescence was more expanded on the stigmatic surface, but the fluorescence pattern was not uniform (Figure 4C). At anther dehiscence (stage 4), the strong green fluorescence was extended to the complete stigmatic surface (Figure 4D). When olive flowers lose petals and anthers (stage 5), the fluorescence labelling was observed only in some regions of the stigmatic surface (Figure 4E). No green fluorescence was observed in the pistil or other flower parts of the control flowers (Figure 4F-J).

**Figure 5:**
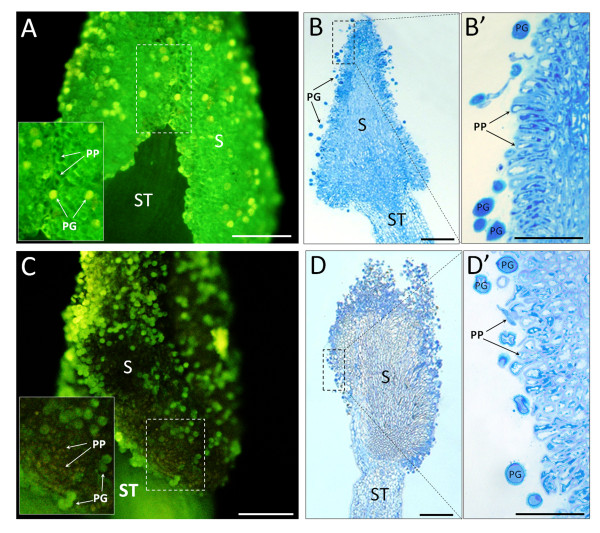
**Ca^2+ ^localization (right panel) and structural features (left panel) of outer stigmatic areas at stages 4 and 5 of olive flower development**. (A) In the flower with dehiscent anthers (stage 4), strong labelling is present throughout the surface of the pollinated stigma. At higher magnification (inset), most of the labelling can be observed as attached to the papillae cells in the form of a thick layer. Yellowish autofluorescence of the pollen grains present on the stigmatic surface is visible. (B and B') The stigmatic surface is composed of externally oriented, vacuolated papillae cells. Numerous pollen grains are present on the stigma. (C) In the pistil from a flower without sepals and petals (stage 5), weak labelling is present in some papillae cells. Yellowish fluorescence is observed in pollen grains attached to the stigmatic surface. (D and D') Degeneration of papillae cells can be observed on the whole stigmatic surface. Numerous pollen grains are still attached to the stigmatic surface. Bars = 100 μm.

**Figure 6:**
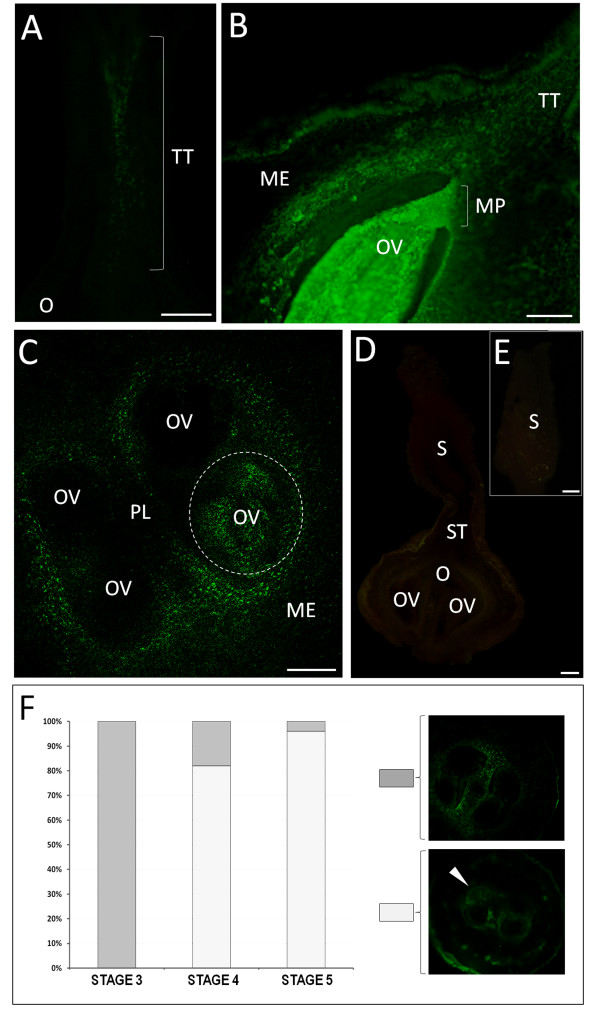
**Ca^2+ ^detection in the internal parts of the pistil from flowers with dehiscent anthers**. (A) In the longitudinally cut style, accumulation of green fluorescence is present in the area of the transmitting tract. (B) In the lower style and ovary, the labelling is located in the transmitting tract and around the loculus; stronger green fluorescence is localized in the whole area of the ovule, beginning from the micropylar region. (C) Transversal section of the ovary. Intense green fluorescence is visible in the areas directly surrounding 2 loculi and only in 1 of the 4 ovules present in the ovary (area marked with the dashed line). The remaining ovules show no signal. (D) Control reaction. In a longitudinally cut pistil that is not injected with Fluo-3, no green fluorescence can be detected in any part of the pistil. (E) Stigma of the control pistil. No green fluorescence is present in the papillae cells or in the attached pollen grains. ME - mesocarp, MP - micropylar region, O - ovary, OV - ovule, PG - pollen grain, PL - placenta, S - stigma, ST - style, TT - transmitting tract. Bars = 100 μm. (F) Graph comparing the percentage of ovaries where none of the ovules showed labelling with those where specific accumulation of Ca^2+ ^only in 1 of the 4 ovules at stages 3, 4, and 5 was indicated.

A more detailed analysis of the localization of the incorporated Fluo-3 AM in the pistil at stages 4 and 5, which are highly significant for sexual plant reproduction events in flowering plants, was also performed (Figure 5 and 6). After anther dehiscence (stage 4), the whole stigma surface showed an intense green labelling observed as associated with the papillae cell surface (Figure 5A, inset). Histochemical staining with methylene-blue confirmed that, at this stage, the stigma was composed of radially oriented papillae cells and was covered with pollen grains, which lend yellowish fluorescence to some areas of the stigmatic surface (Figure 5B and 5B'). The pollen exine always emitted yellowish autofluorescence as it was observed on the negative controls (not shown) (Figure 5A). After petal loss (stage 5), the green fluorescence was much less intense and was localized only in some peripheral parts of the stigmatic surface (Figure 5C). At this stage papillae degeneration occurred, as observed in the methylene blue-stained sections (Figure 5D and 5D').

In the style of the pistil at stage 4, the most intense labelling was located along the transmitting tissue, whereas the remaining stylar tissues showed relatively low staining (Figure 6A and 6B). In the ovary, the strongest signal was detected in the ovule, beginning from the micropylar region (Figure 6B). Remarkable features of the Fluo-3 AM localization pattern were observed in transversally cut ovaries at stage 4 and 5 (Figure 6C). The green fluorescence was observed only in 1 of the 4 ovules present in the ovary (Figure 6C, area marked with the dashed line). Intense labelling was also present in the area directly surrounding the 2 loculi and in the endocarp area. Control reactions carried out by omitting the Fluo-3 AM dye from the injected solution showed no fluorescence in any part of the analyzed pistils (Figure 6D and 6E). The accumulation of fluo3-AM in just one ovule was found in 16 out of 20 ovaries at stage 4 and 19 out of 20 ovaries at stage 5 (Figure 6F).

### Ultrastructural localization of Ca^2+ ^in the stigmatic tissues of the developing pistil

**Figure 7:**
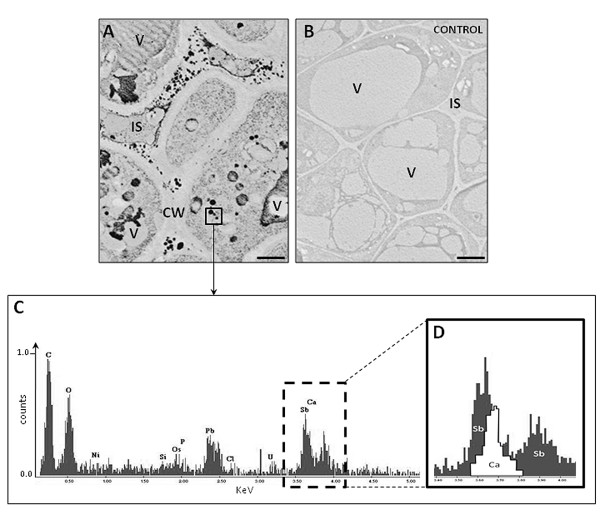
**Identification of Ca^2+ ^in olive pistils by using the pyroantimonate (PA) method**. (A) Numerous electron-dense precipitates are present in the vacuole and in the intracellular spaces of the stigmatic cells (arrows). (B) Negative controls were the pistils fixed without the addition of PA; there is a lack of electron-dense precipitates in the stigmatic cells. V - vacuole. Bars = 1 μm;. (C) Energy dispersive x-ray analysis of the electron-dense deposits present in the ultrathin sections of stigma cells (area marked as square in A). (D) Overlapping peaks of Ca and Sb confirm the identity of calcium antimonite precipitates. The spectrum of the material reveals peaks for Ca and Sb.

To study the subcellular distribution of Ca^2+ ^ions, we used the pyroantimonate method, which is used to localize free and loosely bound calcium. This method revealed many electron-dense precipitates in the cells of the different olive pistil tissues. Precipitates were mainly localized in the large vacuoles and in the intercellular spaces (Figure 7A). In the control sections, where the material was fixed without the addition of pyroantimonate, electron-dense precipitates did not occur (Figure 7B). Energy-dispersive x-ray spectroscopy (EDX)-based analysis of the electron-dense precipitates showed peaks of Sb and Ca (Figure 7C and 7D), confirming that these precipitates included Ca[Sb(OH)_6_]_2_, the reaction product of the pyroantimonate technique.

**Figure 8:**
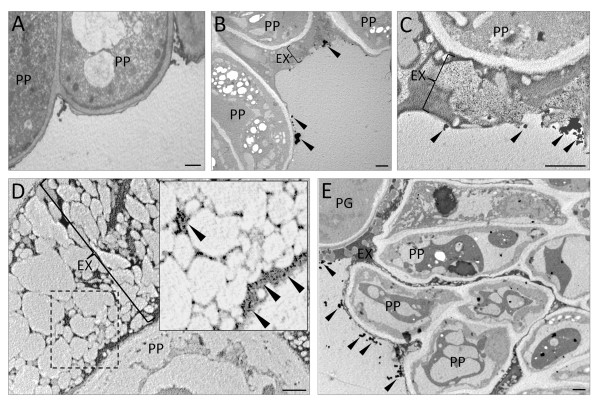
**Subcellular localization of Ca^2+ ^in the stigmatic surface of developing olive pistils**. (A) Stigmatic surface of the pistil enclosed in a green flower bud (stage 1). No electron-dense precipitates can be found in the stigma surface or in the papillae cells. (B) Stigmatic papillae at the beginning of flower opening (stage 2): a few Ca/Sb precipitates are localized on the outer surface of the papilla cell walls (arrowheads). (C) Stigmatic papillae of a completely open flower with turgid anthers (stage 3): thick layer of exudate that has plentiful electron-dense precipitates is present on the outer stigmatic surface. (D) Magnified area of a rich exudate layer (inset, area marked with the dashed line) present on the stigmatic surface at the time of anther dehiscence (stage 4). Numerous, small Ca/Sb precipitates are located exclusively over the electron-dense matrix of the exudates (arrowheads). (E) In the stigma of a flower without petals and anthers (stage 5), Ca/Sb deposits are less abundant and present mainly on the surface of degenerating papillae cells and pollen grains (arrowheads); PG - pollen grain, PP - papillae cell, EX - exudate. Bar = 1 μm.

Particularly interesting was the distribution of precipitates on the stigmatic surface of the developing pistil. In the green flower bud, no detectable Ca^2+ ^ions were observed in the papillae cells as well as at the stigmatic surface (Figure 8A). At the beginning of anthesis (stage 2), we found some electron-dense precipitates on the outer surface of the papilla cells and the stigmatic exudate (Figure 8B). When the flower was open (stage 3), a rich pool of fine and thick precipitates were localized in the papillae exudate layer (Figure 8C). At the time of anther dehiscence, when the exudate was copious, numerous Ca/Sb precipitates were observed over the heterogeneous exudate matrix (Figure 8D). After the loss of petals and anthers (stage 5), the precipitates were present on the surface of papillae cells, which showed distinguishable signs of degeneration (Figure 8E).

## Discussion

Here, we used fluorescence microscopy for the *in situ *localization of Ca^2+ ^ions in intact olive pistils after Fluo- 3 AM injection into inflorescences. Fluo-3 AM, similar to other calcium indicators (like those from the Fura family or Indo-1) must be introduced into the examined cells, and this step is a prerequisite to measure intracellular Ca^2+ ^ions by using microscopy imaging techniques. To introduce this dye into intact pistils, we injected the Fluo-3 solution directly into olive inflorescences. To date, this is the first report on using a Ca^2+^-sensitive dye in the form of an acetoxymethyl ester to follow Ca^2+ ^behaviour in plant reproductive organs. The presence of the dye inside the cells of the olive pistil indicates the following: (1) The amount of dye solution used was sufficient to penetrate the tissues of the inflorescence peduncle, whole flowers, and floral organs. (2) The concentration of Fluo-3 esters introduced into the inflorescence tissues was enough to eliminate the previously reported potential problem of Fluo-3 ester hydrolysis by cell wall hydrolases [27,28].

As far as we know, there are no data in the literature reporting the Ca^2+ ^content in whole pistils during their development in angiosperms. Most of the studies on Ca^2+ ^in pistil tissues focused on the period of full maturity and are frequently restricted to defined parts of the pistil, particularly the stigma and ovary [4,16,21,32].

It is well known that Ca^2+ ^is involved in multiple intracellular and intercellular signalling pathways [2,33]. At the earliest analyzed stage of olive flower development (stage 1), the levels of Ca^2+ ^were quite low. This is probably because buds at this stage are tightly closed and practically isolated from any external biotic and abiotic factors. Furthermore, at this stage, the main task of the flower bud is to complete the growth and maturation of anthers and the pistil. Consequently, the intensity of the signalling events in the stigma of the flower bud is low. As progress in flower development occurred, resulting in gradual petal whitening and flower opening (stage 2), an increase in Ca^2+ ^levels, in parallel with its appearance in the stigma, was observed. At this time of olive flower development, we observed the following: (1) the beginning of exudate production and secretion by papillae cells and (2) accumulation of lipids, pectins, arabinogalactan proteins, and other components in the stigmatic tissues [29,30]. Such increase in the metabolic activity of stigmatic tissues requires intensification of signalling events, in which Ca^2+ ^is thought to be a key player. At this stage of flower development, we showed the accumulation of Ca/Sb precipitates in the vacuoles of the stigma cells as well as in the intracellular spaces between them. The stigmatic surface is the main place for signal exchange between pollen and stigma. Ca^2+ ^ions are more abundant in the receptive stigmas than in the non-receptive surfaces [16,34-36]. The highest levels of Ca^2+ ^accumulation were observed in olive stigmatic tissues at the time of pollination. Because in the olive the stigmatic receptivity is closely related with the pollination time, our results support a positive correlation between the Ca^2+ ^levels in the stigmatic exudates and the receptivity state of the stigma in the olive [30]. Thus, we propose that the grade of fluorescence intensity of the incorporated Fluo-3 AM could be used as a potential marker of the degree of stigma receptivity.

The strong decrease of the Ca^2+ ^pool in the pistil at the last stages of pistil development coincides with the degradation of the stigma tissues. The decay of the stigma is the first step in the flower senescence process, which involves structural, biochemical, and molecular changes that lead to programmed cell death (PCD) [37-39]. Flower senescence is also known to be regulated by several signalling pathways involving Ca^2+^. The presence of Ca^2+ ^in the stigmatic exudate at the end of the anthesis period might suggest that this cation is necessary for the onset of the senescence process [39]. Indeed, Serrano *et al*. [40] reported that at the latest stage of olive flower development, once the stigma was completely brown, papillae cells exhibit PCD symptoms as a result of the incompatibility reaction between pollen and papillae stigma cells. In our opinion and according to our results, the papillae cells death is rather a consequence of their developmental program and the Ca^2+ ^accumulation observed in these cells might be one of the PCD hallmarks during stigma senescence.

Significant changes in the stylar Ca^2+ ^pool were also observed at the time of anther dehiscence (stage 4). The Ca^2+ ^labelling in the style was temporally correlated with the receptive phase of the stigma and pollination, since the stigmatic surface was covered with many pollen grains. It supports the involvement of the transmitting tissue in Ca^2+ ^delivery for pollen tube growth. It is well known that pollen tube growth requires Ca^2+ ^ions from the extracellular environment under both *in vitro *and *in vivo *conditions [22,41]. Indeed, the presence of Ca^2+ ^in the style has been reported in *Petunia hybrida *[18] and in tobacco [19]. The implication of Ca^2+ ^in pollen tube growth and its guidance during the progamic phase has also been reported in other species [7,22,19,42,43]. In already pollinated flowers (stage 5), the stigmatic and stylar pool of Ca^2+ ^decreased significantly in comparison to that in stage 4. The low levels of detectable Ca^2+ ^along the style in the olive at this time of the reproduction course indicate that pollen tube growth through the stylar tissues is already complete.

The most striking features of Ca^2+ ^distribution in the olive pistil were observed in the ovary at the time of pollination (stage 4) and fertilization (stage 5). Ca^2+ ^was observed to specifically accumulate in one of the four ovules present in the ovary, whereas the remaining ovules showed no labelling. This localization pattern was observed in more than 80% of the ovaries at stage 4 and in more than 95% of the ovaries at stage 5. It has been established that the micropyle contains high levels of Ca^2+^, which closely correlate with fertility and serve probably as an attractant for the growing pollen tube [4]. In *Nicotiana *and *Plumbago*, the Ca^2+ ^concentration in the micropylar regions reached the peak when the pollen tube arrives [32,44]. Chudzik and Snieżko [45] proposed that such an accumulation of Ca^2+ ^may serve as a marker of ovule receptivity. Indeed, at stage 4, *in situ *accumulation of ovular Ca^2+ ^was observed to start at the micropylar region. However, the presence of this specific "single-ovular" Ca^2+ ^labelling was still observed at the post-anthesis stage of flower development (stage 5) when most of the flowers were successfully fertilized. According to the previous observations that in olive only 1 or 2 (in exceptional cases) ovules are fertilized [31], we suggest that the observed Ca^2+ ^localization pattern might indicate which ovule will be fertilized or has been already fertilized.

It is well known that post-fertilization events leading to fruit formation include changes in the tissue developmental programs, which implicate a continuous exchange of signals between different types of cells [46]. Ca^2+ ^has been shown to play a crucial role in processes such as egg cell activation [20,47], gamete fusion [20,48], or embryo sac degeneration [44,49]. Given that, we propose that Ca^2+ ^fluorescence can be used as a specific marker of fertilized ovules in multiovular ovaries. However, calcium level could remain high after fertilization of this ovule, so further experiments will be necessary to elucidate which explanation is the correct one.

## Conclusions

This report describes the following for the first time: (i) the dynamics of Ca^2+ ^at the whole organ level during the course of pistil development; (ii) the specific Ca^2+ ^labelling of only one ovule in the ovary, probably the one to be fertilized or already fertilized; (iii) the close relationship between stigma senescence and Ca^2+ ^ions; and (iv) introduction of labelling with Ca^2+^-sensitive dyes as a useful marker of stigma receptivity during the flowering period. Summing up, we propose that the progressive increase of the Ca^2+ ^pool during olive pistil development shown by us reflects the degree of pistil maturity and that Ca^2+ ^distribution at organ level can be used as a marker of fundamental events of sexual plant reproduction occurring in the pistil (Figure 2).

## Methods

### Plant material

Inflorescences were collected during May and June of 2010 and 2011 from *Olea europaea *L. trees, cv. Picual, grown in the province of Granada (Spain). Only perfect flowers (with both pistil and stamens) from 5 selected stages of development were used for the experiments.

Pistils, anthers, petals, and calyces were dissected from flower buds/flowers at these developmental stages, immediately frozen with liquid nitrogen, and stored at -80°C. Additionally, for analytical studies, pistils from different developmental stages were divided into two parts, stigma with style and ovary, by using a razor blade. The material was frozen and stored at -80°C.

### Quantification of Ca^2+ ^content

Ca^2+ ^content was measured using the Calcium Colorimetric Assay Kit (BioVision, Mountain View, CA), and the manufacturer's instructions were followed. In brief, 10 mg of each floral organ (stigma with style, ovary, anther, petal, or calyx) from different developmental stages was homogenized with 50 μl of the Calcium Assay Buffer provided with the kit. Samples were centrifuged at 10000 × *g*, and the supernatant was used for further experiments. According to the manufacturer's instructions, 20 μl of each sample was incubated with the reagents provided with the kit in a 96-well plate. The amount of Ca^2+ ^was measured using the BioRad iMark Microplate Reader (Bio-Rad, Hercules, CA, USA) and was expressed as optical density (OD) at 575 nm in micrograms per well. Controls were prepared for all samples by adding 20 μl of the supernatant and filling up with ultrapure water to the final volume of 150 μl per well. OD of the controls at 575 nm was used as background. The final Ca^2+ ^amounts were calculated according to the manufacturer'sprotocol and are given in μg per μl of the sample. A standard curve was prepared using known amounts of the Ca^2+ ^standard included in the kit. Three independent experiments were performed using material collected during the flowering season of 2010 and 2011 (N = 6). The mean and standard deviation values were calculated and plotted using the SigmaPlot software (Systat, Software, Germany).

### Dye injection

The Ca^2+^-sensitive fluorescent dye Fluo-3 AM (1-mM solution in dimethyl sulfoxide [DMSO]) was purchased from Invitrogen (Molecular Probes, Eugene, OR, USA). The intact inflorescences (length, 2 to 3 cm) just after harvesting from the olive trees were immediately injected with a solution containing the following: 20 μM Fluo-3 AM ester, 0.1% (v/v) Nonidet P-40 (Sigma-Aldricht, St. Louis, MI, USA), and ultrapure water. The Fluo-3 AM ester was added from a stock solution of 1 mM Fluo-3 AM in DMSO. The final DMSO concentration in the incubation solution was approximately 1% (v/v). Injection was done directly into the peduncle of the inflorescence at the site of the cut, as shown in Figure 1A. The whole injection procedure was carried out under the Leica Epifluorescence Stereomicroscope M165FC (Leica Microsystems GmbH, Germany) by using a micro-syringe (volume, 200 μl) and a fine needle (diameter, 60 μm) (Bionovo, Legnica, Poland). Into each inflorescence, 100 μl of dye solution was injected. Control samples were injected with 100 μl of solution containing 1% DMSO (v/v), 0.1% Nonidet P-40 (v/v), and ultrapure water. Inflorescences were incubated for 2 h at room temperature in the dark in petri dishes that contained filter paper soaked with ultrapure water. Flower buds and flowers located nearest to the injection site were dissected from the inflorescences and analyzed using microscopy as whole or longitudinal or transversal sections. Ten buds/flowers from each developmental stages of two consecutive flowering seasons have been used to be analyzed.

### Light microscopy

The pistils were fixed in 4% paraformaldehyde (w/v) and 2% glutaraldehyde (v/v) prepared in 0.1 M cacodylate buffer (pH 7.5) at 4°C overnight. After fixation, the material was washed several times in cacodylate buffer, dehydrated in an ethanol series, and embedded in Unicryl resin at -20°C under UV light. Semi-thin (1 μm) sections were obtained using a Reichert-Jung Ultracut E microtome. The sections were placed on BioBond-coated slides and stained with a mixture of 0.05% (w/v) methylene blue and 0.05% (w/v) toluidine blue in order to analyze the histological features of the pistil at each developmental stage [50]. Observations were carried out using a Zeiss Axioplan (Carl Zeiss, Oberkochen, Germany) microscope. Micrographs were obtained using a ProGres C3 digital camera with the ProGres CapturePro 2.6 software (Jenoptic, LaserOptic Systems GmbF, Germany).

### Epifluorescence and confocal laser scanning microscopy

Fluo-3 fluorescence was monitored after excitation with light of 460-500 nm by using an epifluorescence stereomicroscope (Leica M165FC; Leica Microsystems, Bensheim, Germany) equipped with a digital camera controlled by the Leica Imaging software (Leica Microsystems, Bensheim, Germany). The emitted fluorescence was detected at wavelengths above 510 nm. Autofluorescence (mainly due to the presence of chlorophyll and other pigments and secondary metabolites) was isolated and displayed in red. High-resolution images of Fluo-3 fluorescence inside the pistils' tissues were obtained using a Nikon C1 confocal microscope (Nikon, Japan) with an Ar-488 laser source and different levels of magnification (4× to 20×). Small pinhole sizes (30 μm) were used in combination with low-magnification, dry objectives. Optical sections were captured as Z-series images and processed using the software EZ-C1 Gold version 2.10 build 240 (Nikon). The fluorescent signal was obtained exclusively in the range of 510-560 nm emission wavelengths and was recorded in green.

### Ultrastructural localization of Ca^2+^

Ca^2+ ^localization was cytochemically analyzed in pistil tissues by using the pyroantimonate method of Rodríguez-Garcia and Stockert [51]. Pistils were fixed for 24 h in cold (4°C) fixative solution consisting of 5% (w/v) potassium pyroantimonate [(K_2_H_2_Sb_2_)7·4H_2_O] and 2% (w/v) osmium tetroxide at pH 7.5. After fixation, pistil tissues were dehydrated in an ethanol series and embedded in Epon resin. Ultrathin sections were obtained using the Ultracut microtome (Reichert-Jung, Germany) and mounted on 200-mesh formvar-coated nickel grids. Pistils fixed identically, but in the absence of pyroantimonate, were used as controls. Observations were carried out using a JEM-1011 transmission electron microscope (JEOL, Japan).

Pyroantimonate precipitates present in ultrathin sections on carbon-coated nickel grids were examined under a STEM PHILIPS CM20 microscope equipped with an energy-dispersive x-ray (EDX) detector at the Scientific Instrumentation Centre of Granada University, Granada, Spain.

## List of abbreviations

AM: acetoxymethyl; DMSO: dimethyl sulfoxide; OD: optical density; PCD: programmed cell death.

## Authors' contributions

MIRG conceived the study. JDA and AJC supervised the experiments. KZ and JDR carried out the experiments and contributed equally to this paper. CS carried out the histochemical studies. The six authors discussed the results and prepared the manuscript. All authors read and approved the final manuscript.
